# A New Comparative-Genomics Approach for Defining Phenotype-Specific Indicators Reveals Specific Genetic Markers in Predatory Bacteria

**DOI:** 10.1371/journal.pone.0142933

**Published:** 2015-11-16

**Authors:** Zohar Pasternak, Tom Ben Sasson, Yossi Cohen, Elad Segev, Edouard Jurkevitch

**Affiliations:** 1 Department of Plant Pathology and Microbiology, the Robert H. Smith Faculty of Agriculture, Food and Environment, the Hebrew University of Jerusalem, Rehovot, Israel; 2 Department of Applied Mathematics, Holon Institute of Technology, Holon, Israel; University Hospital of Heidelberg, GERMANY

## Abstract

Predatory bacteria seek and consume other live bacteria. Although belonging to taxonomically diverse groups, relatively few bacterial predator species are known. Consequently, it is difficult to assess the impact of predation within the bacterial realm. As no genetic signatures distinguishing them from non-predatory bacteria are known, genomic resources cannot be exploited to uncover novel predators. In order to identify genes specific to predatory bacteria, we developed a bioinformatic tool called DiffGene. This tool automatically identifies marker genes that are specific to phenotypic or taxonomic groups, by mapping the complete gene content of all available fully-sequenced genomes for the presence/absence of each gene in each genome. A putative ‘predator region’ of ~60 amino acids in the tryptophan 2,3-dioxygenase (TDO) protein was found to probably be a predator-specific marker. This region is found in all known obligate predator and a few facultative predator genomes, and is absent from most facultative predators and all non-predatory bacteria. We designed PCR primers that uniquely amplify a ~180bp-long sequence within the predators’ TDO gene, and validated them in monocultures as well as in metagenetic analysis of environmental wastewater samples. This marker, in addition to its usage in predator identification and phylogenetics, may finally permit reliable enumeration and cataloguing of predatory bacteria from environmental samples, as well as uncovering novel predators.

## Introduction

Predation between microorganisms affects major ecological processes on a global scale. Micro-predation (as defined by the destruction of a viable microbial cell) is an important component of the marine microbial loop, through the consumption of bacteria and archaea by protists and their lysis by phages [1]. Protists and phages also have strong effects on freshwater microbial food webs by contributing to prokaryotic mortality [2,3]. In soil, protozoa enhance nitrogen mineralization, leading to increased plant nitrogen uptake and plant growth [4]. Although much less is known on soil phages, they may be present at high densities, potentially contributing to microbial turnover [5,6]. In addition to protozoa and phages, bacteria can also perform predation on one another. Predatory bacteria include obligate and facultative predators, which together can prey on a large variety of other bacteria [7]. Moreover, they have the capacity to attack and consume a variety of multidrug-resistant clinical strains, maintaining their predation regardless of prey antimicrobial resistance [8]; hence, they might be used as therapeutic agents where antimicrobial drugs fail. Although predators are distributed between many of the higher phyla, their currently-known total diversity amounts to only about 20 genera [7]. This dearth stems from our inability to identify predatory interactions from microscopic observation of natural samples and by the limitation of culture-based characterization by the growth requirements of both prey and predator.

Recently we developed an approach by which predator-enriched or predator-depleted protein families were identified by comparing the proteomes of predatory vs. non predatory bacteria, enabling the detection of predatory capacities in full genomes [9,10]. Nevertheless, no genes have been found to be unique to predators. With such a tool at hand, it may become possible to further screen genomes for potential predatory abilities and also assess the abundance of bacterial predators in the environment, an essential step toward understanding the effects of bacterial predation in nature. Now, with the growing availability of whole genome data, new methods have emerged for systematically finding optimal genetic markers to distinguish between phylogenetic or phenotypic groups [11,12,13,14]. However, none of the existing methods enabled us to find enriched genes in thousands of genomes simultaneously in a user-friendly and efficient manner. To that end, we designed a novel bioinformatics tool, and used it to find, for the first time, a predation marker gene; we further investigated this gene to design PCR primers for identification and analysis of predatory organisms in monoculture. Finally, we showed that the gene specifically detects predatory bacteria in environmental samples.

## Materials and Methods

### Software development

A novel software, DiffGene, takes advantage of the orthologous gene cluster table created and maintained by the microbial genome database (MBGD) [15] and freely available at http://mbgd.genome.ad.jp/htbin/view_arch.cgi. This table is updated twice a year, and is arranged so that each row in the table is an orthologous cluster (i.e. the same gene) and each column is a genome. The ortholog identification and grouping procedure, called DomClust, is described in full elsewhere [16]; in short, it takes as input all-against-all protein BLAST similarity data and classifies genes based on subsequent hierarchical clustering with UPGMA [17]. During clustering, it detects domain fusion or fission events, splits clusters into domains (if required), and then splits the resulting trees such that intra-species paralogous genes are divided into different groups so as to create plausible orthologous groups. Next, a second procedure called DomRefine [15] improves domain-level clustering using multiple sequence alignment information, and a third (MergeTree) [18] adds new genomes to the table.

The raw MBGD data, as is often the case for such bulky datasets, is too large and complex to be handled on a personal computer. In DiffGene, these data are automatically cleaned, un-needed and redundant data are deleted, and all gene occurrences are transformed from gene names into binary data so that each datapoint in the table only contains either a one or a zero (the gene is present or absent in the genome, respectively). This reduces the file size by two orders of magnitude, and enables efficient algorithm usage. Genomes are then assigned into two groups, ‘present’ and ‘absent’; for each gene, the proportion of genomes in each group which contain that specific gene is calculated. Different fraction threshold levels for the two groups can be assigned so the output of the analysis contains only genes which appear in at least the indicated fraction of ‘present’ genomes and at most in the indicated fraction of ‘absent’ genomes. The software is freely available at http://departments.agri.huji.ac.il/plantpath/jurkevitch/ej-software.html.

### Search for predation-specific marker

Predators are phylogenetically diverse but share some phenotypic or ecological traits (S1 Table). We employed DiffGene to find genes enriched in predatory compared to non-predatory genomes. DNA and protein sequences of the most-discriminating gene were taken from the NCBI RefSeq database, representing predatory and non-predatory bacteria, as well as eukaryotic organisms. Protein sequences were aligned using MUSCLE [19] and a maximum-likelihood phylogenetic tree was constructed in MEGA6 [20].

### Experimental confirmation of specificity

DNA sequences of the candidate marker gene were also aligned and manually inspected to find potential primers for a PCR reaction. The most predator-specific primers were named TDO-F (5’-TAYGARYTVTGGTTYAARCARAT-3’) and TDO-R (5’-GGMGTCATSSTYTCVA-3’) (for nucleotide ambiguity codes, see [21]). DNA to be used as PCR template was extracted with PowerSoil isolation kit (MoBio laboratories, Carlsbad, CA) from pure cultures of three predators (*Bdellovibrio bacteriovorus* HD100, *Bdellovibrio exovorus* JSS, and *Peredibacter starrii* A3.12) and five non-predators from various phyla (*Escherichiacoli* ML35, *Pseudomonas* sp., *Flavobacterium* sp., *Burkholderia* sp. and *Photobacterium* sp.). PCR reactions were performed using 12.5 ul master mix (0.1 U/μl Taq Polymerase, 500 μM dNTP each, 20 mM Tris-HCl (pH 8.3), 100 mM KCl, 3 mM MgCl2), 8.5 ul double-distilled water, 2 ul of each primer and 2 ul of template DNA; amplification conditions were 95°C for 5 min, followed by 36 cycles of 95°C for 30 sec, 50°C for 30 sec, 72°C for 30 sec, and a final stage of 72°C for 7 min. The same primers were used to assess the predator communities in four environmental samples from a wastewater treatment plant. DNA extraction and PCR amplification were as before, and MiSeq next-generation sequencing (Illumina, USA) was performed as previously described [22]. Sequences were processed in MOTHUR v1.34 [23]: quality, length and adapter trimming were performed on the forward (non-paired) reads as previously described [24], resulting in >50,000 reads per sample with a uniform length of 184 nucleotides per read. Datasets were deposited at the MG-RAST database (http://metagenomics.anl.gov/linkin.cgi?project=13062) under accession numbers 4624348.3–4624351.3. Sequences sharing 97% identity were clustered into the same operational taxonomic unit (OTU) and the representative sequence from each OTU was phylotyped using BLAST. The representative sequences of the 100-most abundant OTUs, along with marker gene sequences from predators and non-predators, were used for creating a phylogenetic tree as above.

## Results

### Multi-locus typing of all predatory bacteria

Of the 2286 complete (non-draft) bacterial genomes available on the MBGD dataset at the time of analysis, 14 belonged to known predator species (S1 Table) and the other 2272 were considered non-predators. DiffGene analysis discovered several genes which were quite specific to either genomes of predators or of non-predators, but none of them was 100% specific to either group. Combining three of these genes–*kynA*, *waaL* and *gntR* (Table 1)–such that a genome would be considered ‘predatory’ if it contained the former two and lacked the latter, resulted in correct classification of 14/14 (100%) of the predators and 2255/2272 (99.3%) of the non-predators. It bears noting that since one bacterial class, namely delta-proteobacteria, was over-represented in the 'predatory' genomes, the three-gene set could in fact have been indicative not of predators but of delta-proteobacteria. However, this is unlikely because of the 50 'non-predatory' delta-proteobacterial genomes, five contained *kynA*, five *waaL*, and 39 *gntR*, thus all were correctly identified as non-predatory (except for *Anaeromyxobacter* which is a potential predator).

**Table 1:** Abundance of marker genes in genomes of predatory and non-predatory bacteria.

Gene	Rep. accession	Predators	Non-predators
[*kynA*] Tryptophan 2,3-dioxygenase	NP_968676	14/14 = 100%	302/2272 = 13%
[*waaL*] O-antigen ligase	NP_968553	14/14 = 100%	434/2272 = 19%
[*gntR*] Transcription regulator	YP_004789625	0/14 = 0%	1221/2272 = 54%

Rep., representative.

### Marker gene for obligatory predatory bacteria

**Fig 1:**
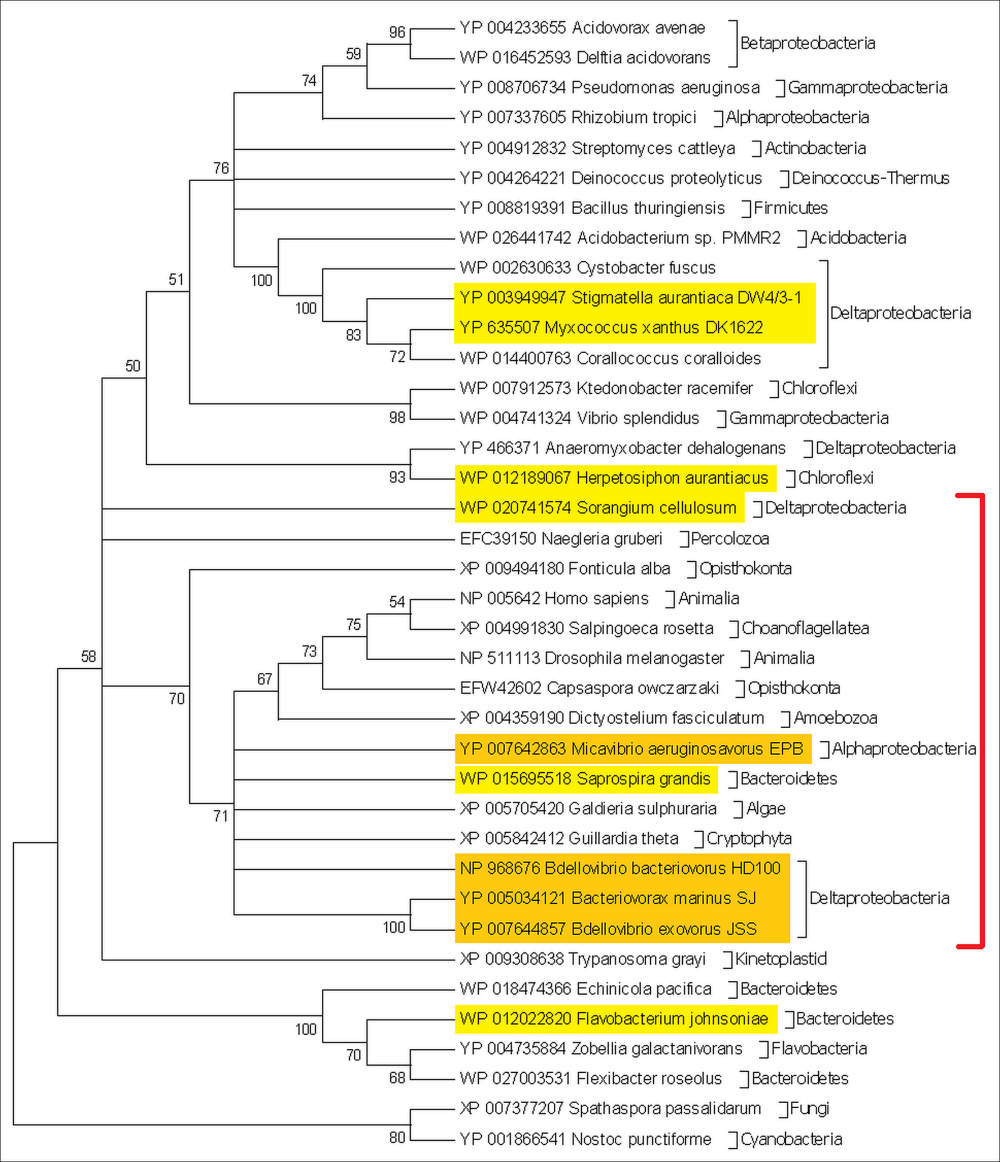
Maximum-likelihood phylogenetic tree of the tryptophan 2,3-dioxygenase protein. The percentage of trees in which the associated taxa clustered together (out of 100 bootstraps) is shown next to the branches; branches with <50% were collapsed. Obligate bacterial predators are marked orange, facultative yellow. Red line indicates genomes with the ~60 amino acid-long insert.

The top marker gene, *kynA* (encoding tryptophan 2,3-dioxygenase—TDO) was found in 100% (14/14) of predator and 13.3% (302/2272) of non-predators genomes. Of the 302 non-predatory genomes containing *kynA*, five were delta-proteobacteria (out of 50 delta-proteobacteria in the database). Protein sequence analysis of *kynA* representatives revealed that sequences from all obligate and two facultative predatory bacteria, as well as eukaryotic organisms, although phylogenetically extremely diverse, are longer than the non-predatory bacterial ones, as is reflected in their proximity in the TDO phylogenetic tree (Fig 1). The main feature distinguishing the sequences from the two groups is a segment ~60 amino acids long (S1 Fig), absent from all non-predatory bacteria (0%, 0/2272) and most facultative predatory bacteria. This entire segment is a long alpha-helix, which is hydrophilic (mean±SD Kyte-Doolittle hydropathy of -0.76±0.75) and charged (-1.84 at pH = 7). TDO, by itself in bacteria and together with indoleamine 2,3-dioxygenase (IDO) in the mammalian liver, catalyses the first and rate-limiting step in the kynurenine pathway, converting L-tryptophan to N-formyl-kynurenine [25]. The next step in this pathway is converting the N-formyl-kynurenine to formyl-anthranilate (by the enzyme kynureninase) or to L-kynurenine (by the enzyme kynurenine formamidase). Further investigation revealed that all eukaryotes, bacterial non-predators and facultative predators which possessed the *kynA* gene, also possessed the genes required for completing the kynurenine pathway, with the genes for kynureninase and/or kynurenine formamidase usually adjacent to the *kynA* gene. However, in all obligate bacterial predators, no other gene belonging to this pathway was found.

### Validation of TDO sequence specificity in predators

**Fig 2:**
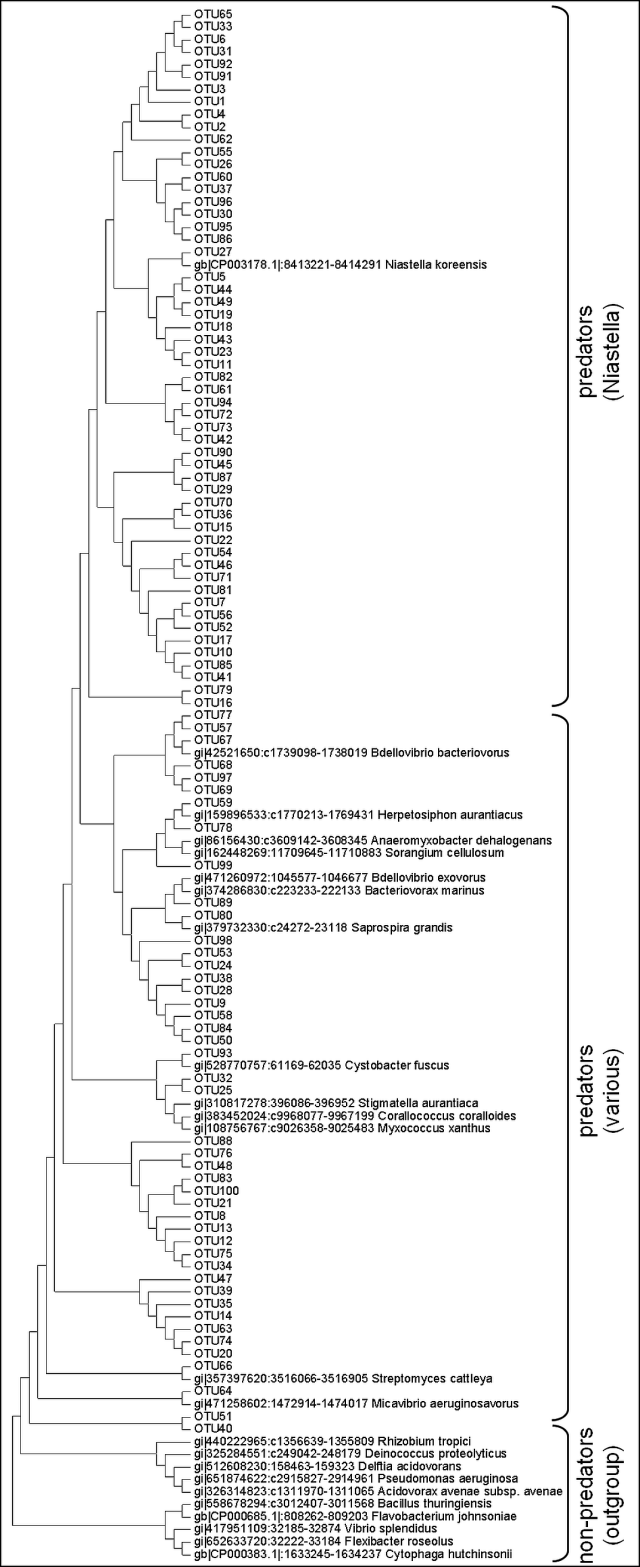
Maximum-likelihood phylogenetic tree of the representative sequences of the 100-most abundant OTUs in the metagenetic analysis, including representative TDO sequences from known predatory and non-predatory bacteria. The bootstrap consensus tree inferred from 100 replicates is taken to represent the evolutionary history of the taxa analyzed. Wastewater OTU names are according to abundance, i.e. OTU1 is the most abundant OTU in the environmental samples, OTU2 is the second-most abundant, and so on. Known sequence names include GI accession, coordinates within the genome, and species name.

The nucleotide sequences of the TDO gene from representative species were aligned, and PCR primers were manually designed to selectively amplify the specific sequence in the TDO genes of predators. PCR amplification was performed on DNA extracted from cultured representative predatory and non-predatory bacteria. A ~180 bp-long PCR product was obtained from the genomes of all predators, whereas no amplicon was obtained from any of the non-predators (S2 Fig). The amplicon was subsequently sequenced and validated to be part of the TDO gene. To further test the specificity of the *kynA* PCR primers, metagenetic next-generation sequencing was conducted on four environmental wastewater samples. Such samples include many more genomes than the databases upon which in-silico analysis was performed, thus providing a more stringent test for specificity. The sequences in each wastewater sample were clustered into operational taxonomic units (OTUs), where all the sequences in a single OTU are at least 97% similar to each other. All samples contained between 67–91 OTUs, and all four rarefaction curves reached saturation after ~30,000 reads (S3 Fig), implying that all the diversity in the samples was detected. When comparing both known and wastewater TDO sequences in a phylogenetic tree, all known non-predators formed an outgroup; of the 100-most abundant OTUs in the wastewater samples, comprising >99% of all sequences, none were phylogenetically close to any known non-predator (Fig 2). This result was also observed when BLASTing each environmental TDO sequence against the NCBI database. All TDO wastewater OTUs belonged to two broad phylogenetic groups: the first, similar to many of the known predators, and the second, similar only to a single known bacterium, *Niastella koreensis* (Fig 2). The seven most highly-abundant environmental OTUs (named OTU1-OTU7 in Fig 2) encompassed 65% of all environmental sequences, and all of these belonged to the "*Niastella*-like" group.

## Discussion

Predatory interactions between bacteria are difficult to detect and therefore it is difficult to address their effect in nature. Further complicating the assessment of their diversity, distribution and abundance is the lack of genetic markers so that metagenomic data are almost blind in ascertaining their status in environmental samples. In order to overcome these difficulties, we have developed DiffGene, a software that allows a quick and easy characterization of marker genes for microbial groups, as long as sequenced genomes representing the groups of study are available. Using all available fully-sequenced genomes, marker genes are optimally chosen so that their presence or absence (rather than their sequence or abundance) is an indication of the genomes’ grouping. Finding marker genes for specific microbial organisms, grouped by either phenotype or taxonomy, can be a challenging task. In our previous work [9,10] proteome similarity matrices were used on a much smaller scale to detect genes unique to bacterial predators. The gene matrix, being based on gene abundance rather than absence/presence data, resulted in less-specific markers but revealed specific genomic properties of most predators. Most strikingly, several genes from the mevalonate pathway (isoprenoid biosynthesis) were highly enriched in most predators; however, while useful for screening cultured organisms, its absence from the genomes of a few predators may lead to false negative results. Its further presence in Archaea and in some non-predatory bacteria would make these genes poor markers for predators in environmental samples. Appropriately, the absence/presence matrix developed here hardly marked any mevalonate pathway genes as highly predator-specific.

We found the *kynA* gene, encoding tryptophan 2,3-dioxygenase (TDO), to be the most predator-specific gene. TDO is the first enzyme in the degradation of tryptophan pathway, and was previously found among the predator-enriched complement of genes [9]. Surprisingly, no other gene belonging to this pathway was found in the genomes of obligate predatory bacteria, whereas all other genomes containing *kynA* contained the genes necessary to complete the tryptophan degradation. This suggests that obligate predators either degrade L-tryptophan by another, unknown pathway, or that they do not catabolize L-tryptophan at all, instead using the N-formyl-kynurenine produced by the TDO for another, unknown purpose. The second most potent marker for predators, the *WaaL* protein, is also a metabolic gene. It catalyzes a critical step in lipopolysaccharide synthesis as it establishes the glycosidic bond of a sugar at the proximal end of the undecaprenyl-diphosphate (Und-PP)-O-antigen with a terminal sugar of the lipid A-core oligosaccharide [26]. The widely distributed regulatory protein *GntR*, absent from the predators, is involved in amino acids, sugars, fatty acids and alkylphosphonate metabolism and pyridoxal phosphate-dependent aminotransfer [27]. It has been proposed that this family goes back to the last common universal ancestor [27], implying that, for an unknown reason, it is selectively lost in predatory bacteria. Using these three genes as a multi-locus sequence typing scheme yielded near-perfect results, except for 17 out of 2272 ‘non-predators’ which were classified as ‘predators’; it is possible that at least some of these 17 are indeed previously-undetected predators. Most of them are suspected predators such as *Flexibacter* [28,29] and *Anaeromyxobacter* [30] or gliding heterotrophs (e.g. *Owenweeksia*, *Fluviicola*, *Echinicola*) that may yet prove to be predatory.

The finding of a predation-specific marker advances the research of predatory bacteria in three important aspects: first, eliminating—for the first time—the need for a laborious *in-vitro* predator and prey co-culture in order to ascertain whether a species is indeed predatory. This was confirmed using cultured strains. Second, shedding new light on predator phylogenetics, since the TDO gene obviously bears some significance for the predatory lifestyle and may thus prove more phylogenetically informative than the ubiquitous 16S rRNA gene. Third, applying the predator-specific TDO PCR primers in real-time PCR and next-generation metagenetic sequencing applications may finally permit reliable enumeration and cataloguing, respectively, of predators from environmental samples, as well as uncovering novel predatory bacteria. In the tested wastewater samples, none of the 100-most abundant OTUs (encompassing >99% of all sequences) could be traced to known non-predators; while these OTUs could theoretically hail from unknown non-predators, it is more likely that these findings confirm that the approach is indeed predator-specific. Interestingly, our preliminary analysis of wastewater samples revealed that a large portion of the predator OTUs and sequences had no known relatives except *Niastella koreensis*, a gliding bacterium of the phylum Bacteroidetes which is most closely related to the genera *Flexibacter*, *Cytophaga* and *Flavobacterium* [31]; as it happens, all three of these genera contain species which are known predators [7,29], leading us to assume that *Niastella koreensis*, as well as the entire *"Niastella*-like" group apparent in Fig 2, are indeed predators. The many unrecognized predator OTUs suggest that predatory bacteria are unrepresented in culture collections.

Surprisingly, the predator-specific TDO protein is most similar to the eukaryotic one. It has long been thought that, 1.5 billion years ago, the eukaryotic cell originated from a merger of two prokaryotes, an archaeal host and a bacterial endosymbiont [32]; then, during the evolutionary transition from an endosymbiont to an organelle, the bacterium transferred some of its DNA to the host chromosomes [33]. However, since prokaryotes are unable to perform phagocytosis, the means by which the endosymbiont originally entered its host is an enigma. Davidov and Jurkevitch [34], based on [35], suggested that this process was facilitated by a predatory bacterium which penetrated and replicated within the host periplasm, and later became the mitochondria. In a previous study [9], we found that the mevalonate pathway, which is the isoprenoid synthesizing mechanism in eukaryotes (but not in most bacteria), is also strongly enriched in predators. Together, the mevalonate and TDO data add a tantalizing phylogenetic clue to a possible connection between predatory bacteria and eukaryotic evolution.

The genome-centered approach developed in DiffGene and the flexibility awarded to alter the required specificity of markers can provide strict specificity allowing for unambiguous identification of membership in a particular bacterial group at the cost of potential false negative. In contrast, more relaxed specificity settings enable the assignment of microorganisms to groups even if they may not all contain all the marker genes, thus discovering genomes enriched for particular functions and pathways. The approach developed here may be useful for additional purposes as well. Many microbial pathogens are characterized using multi-locus typing, where up to 16 genes are selected as molecular markers and compared between isolates, either by their presence/absence or sequence [36]. Nevertheless, this approach can lead to erroneous typing due to the genes being non-representative [37] and requires many marker genes per group in order to verify the isolates’ membership. Furthermore, genetic markers are often selected *ad hoc*, using too few reference genomes and/or manual inspection of the results [38]. Applying DiffGene for marker search in this context may help overcome such limitations.

## Ethics statement

This study did not involve humans, human data or animals; therefore, no ethics approval was required.

## Consent

This article is not a prospective study involving human participants, and does not contain individual clinical data; therefore, no consent for publication was required.

## Supporting Information

S1 Fig Multiple alignment of the region specific to obligate bacterial predators and eukaryotes of the tryptophan 2,3-dioxygenase protein. Top, obligate bacterial predators and eukaryotes; bottom, non-predatory and facultative predatory bacteria. Residues are colored according to the ClustalX color scheme: blue = hydrophobic, green = polar, magenta = negatively charged, red = positively charged, pink = cysteine, orange = glycine, yellow = proline.
(TIF)

S2 Fig PCR amplification using predator-specific TDO primers on DNA extracted from cultures of predatory and non-predatory bacteria. L, ladder (100bp); 1, *Bdellovibrio bacteriovorus* HD100; 2, *Bdellovibrio exovorus* JSS; 3, *Peredibacter starrii* A3.12; 4, *Escherichia coli* ML35; 5, *Pseudomonas* sp.; 6, *Flavobacterium* sp.; 7, *Burkholderia* sp.; 8, *Photobacterium* sp.; C, negative control.
(TIF)

S3 Fig Rarefaction curves of bacterial communities at 97% sequence similarity level in the four samples from various areas of the wastewater treatment plant. (TIF)

S1 Table Predatory bacteria analyzed in this study. (DOCX)
