# Supplementary figures and images for: A New Comparative-Genomics Approach for Defining Phenotype-Specific Indicators Reveals Specific Genetic Markers in Predatory Bacteria
Source: PLoS One. 2015 Nov 16;10(11):e0142933. doi: 10.1371/journal.pone.0142933 (PMC4646340; doi:10.1371/journal.pone.0142933)

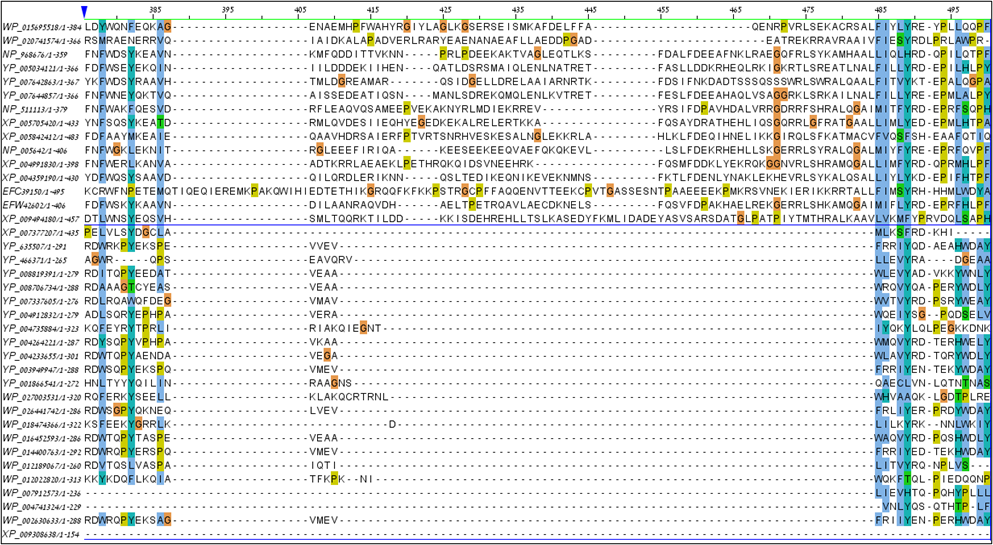

Supplement: S1 Fig — Top, obligate bacterial predators and eukaryotes; bottom, non-predatory and facultative predatory bacteria. Residues are colored according to the ClustalX color scheme: blue = hydrophobic, green = polar, magenta = negatively charged, red = positively charged, pink = cysteine, orange = glycine, yellow = proline. (TIF) [file pone.0142933.s001.tif]

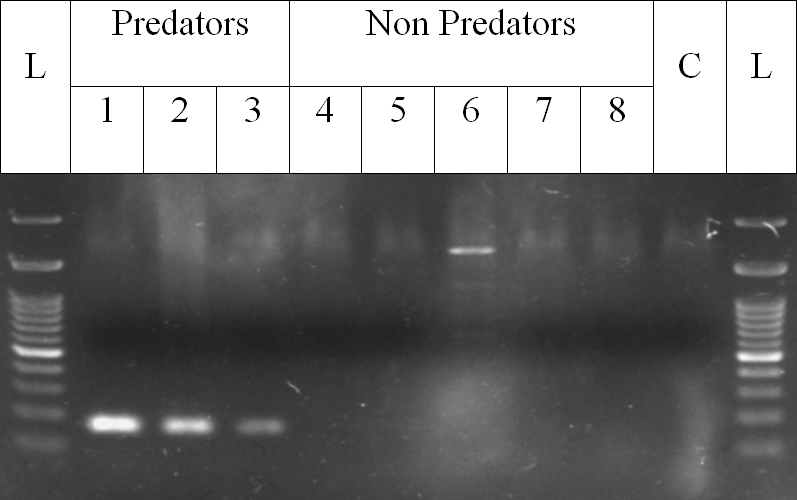

Supplement: S2 Fig — L, ladder (100bp); 1, Bdellovibrio bacteriovorus HD100; 2, Bdellovibrio exovorus JSS; 3, Peredibacter starrii A3.12; 4, Escherichia coli ML35; 5, Pseudomonas sp.; 6, Flavobacterium sp.; 7, Burkholderia sp.; 8, Photobacterium sp.; C, negative control. (TIF) [file pone.0142933.s002.tif]

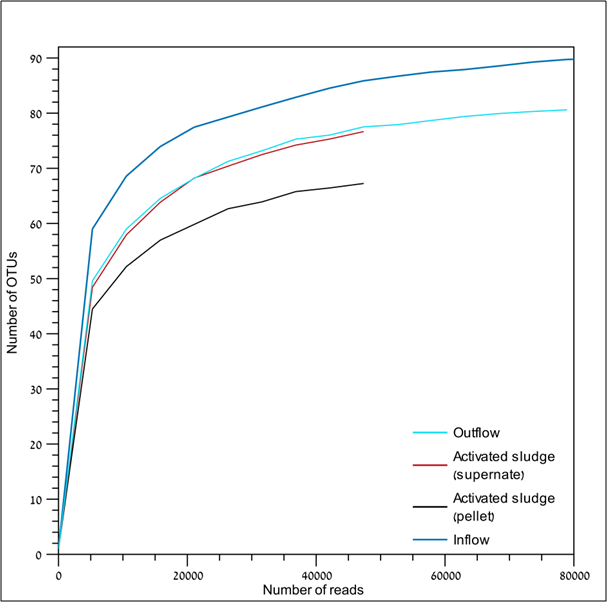

Supplement: S3 Fig — (TIF) [file pone.0142933.s003.tif]
