# Supplementary material for: A New Comparative-Genomics Approach for Defining Phenotype-Specific Indicators Reveals Specific Genetic Markers in Predatory Bacteria
Source: PLoS One. 2015 Nov 16;10(11):e0142933. doi: 10.1371/journal.pone.0142933 (PMC4646340; doi:10.1371/journal.pone.0142933)
Supplement: S1 Table — (DOCX) [file pone.0142933.s004.docx]

Supplementary table S1. Predatory bacteria analyzed in this study.

| **Species name** | **Genome accession** | **Taxonomic affiliation** | **Mode of predation** | **Obligate/**  **facultative** |
| --- | --- | --- | --- | --- |
| *Cytophaga hutchinsonii* | NC_008255 | Bacteroidetes | Epibiotic | Facultative |
| *Flavobacterium johnsoniae* | NC_009441 | Bacteroidetes | ? | Facultative |
| *Saprospira grandis* | NC_016940 | Bacteroidetes | Wolf pack? | Facultative |
| *Herpetosiphon aurantiacus* | NC_009972 | Chloroflexi | Wolf pack? | Facultative |
| *Micavibrio aeruginosavorus* | NC_020812 | α-proteobacteria | Epibiotic | Obligate |
| *Bdellovibrio bacteriovorus* | NC_005363 | δ-proteobacteria | Periplasmic | Obligate |
| *Myxococcus xanthus* | NC_008095 | δ-proteobacteria | Wolf pack | Facultative |
| *Stigmatella aurantiaca* | NC_014623 | δ-proteobacteria | Wolf pack? | Facultative |
| *Sorangium cellulosum* | NC_010162 | δ-proteobacteria | ? | Facultative |
| *Bdellovibrio exovorus* | NC_020813 | δ-proteobacteria | Epibiotic | Obligate |
| *Bacteriovorax marinus* | FQ_312005 | δ-proteobacteria | Periplasmic | Obligate |
